# A comparative analysis of the outcomes of Rezūm therapy based on prostate size: a four-year retrospective analysis

**DOI:** 10.1007/s00345-025-05793-0

**Published:** 2025-07-17

**Authors:** Mohamed Samir, Mahmoud Ahmed Mahmoud, Hossam Abdelsamie Abdelmonem, Mohamed Elrefaie, Khaled Abdelsattar Gad

**Affiliations:** 1 Department of Urology, Faculty of Medicine, Ain Shams University, Cairo, Egypt; 2 Urology Department, Armed Forces College of Medicine, Cairo, Egypt; 3 Military Medical Academy, Cairo, Egypt

**Keywords:** Rezūm, BPH, Small prostate, Large prostate

## Abstract

**Objective:**

To assess the safety and efficacy of Rezūm therapy in small versus large prostates through a four-year follow-up.

**Patients and methods:**

One-hundred seventy patients who underwent Rezūm therapy were retrospectively included and divided into 2 equal groups, small (˂ 80gm) and large prostate groups (80-120 gm). Both groups were assessed preoperatively and at 3, 12, 24, 36, and 48 months post-procedure for operative time, number of vapor injection, catheterization time, hospital stay and post-procedure: prostatic size, prostate-specific antigen (PSA), post-void residual (PVR), maximum urinary flow rate (Qmax), International Prostate Symptom Score (IPSS), quality of life (QoL), International Index of Erectile Function (IIEF) and complications.

**Results:**

The number of vapor injections was significantly higher in the large prostate group compared to the small prostate group (*p* < 0.001). Operative time and catheter duration were significantly longer in the large prostate group (*p* < 0.001), while hospital stay was similar between groups (*p* = 0.407). At 48 months post-Rezūm, both groups demonstrated statistically significant improvement in all measured outcomes (Qmax, IPSS, PVR, QoL, PSA, IIEF, and prostate size) (*p* < 0.05). Regarding postoperative complications, there were no significant differences between the groups except that the need for retreatment was significantly higher in the large prostate group.

**Conclusion:**

Rezūm therapy offers a safe and effective treatment option for both small and large prostates with sustained improvement over four years. However, patients with large prostates exhibited a higher retreatment rate (15.2%).

## Introduction

In patients with benign prostatic hyperplasia (BPH) and gland size > 80 cc, the historically preferred treatment with the greatest durability and efficacy has been simple prostatectomy [1]. However, this procedure is associated with high morbidity, including a higher rate of postoperative complications, as well as longer catheterization and hospital stay [2]. As a result, multiple investigators have assessed the alternative resection or enucleation treatment options with comparable results [3–8].

Rezūm therapy utilizes thermal energy generated by water vapor to ablate prostatic tissues [9]. In 2015, It gained approval from the U.S. Food and Drug Administration (FDA) based on results from a pivotal study (NCT01912339) [10]. However, the Canadian Urological Association (CUA) and American Urological Association (AUA) still don’t recommend its use for patients with large prostates (> 80cc) [11]. Similarly, the European Urological Association (EAU) states that Rezūm therapy use in large prostates requires more randomized controlled studies before it can be recommended [12].

High-quality studies investigating its application in large prostates remain limited, and some centers have confined Rezūm therapy indications for volumes of 30–80 cc [13]. Despite initial promising results, most existing trials provide only short-term follow-up [12, 14, 15]. The aim of our study was to evaluate the safety and efficacy of Rezūm therapy in treatment of patients with large prostates (80 −120gm) via a retrospective trial with a 4-year follow-up period.

## Patients and methods

### Sample size

This was calculated using the G Power 3.1.9.4 program, with an alpha error set at 0.05 and the study power at 80%. According to Garden et al. (2021), the IPSS improved in both the small and large prostate groups, with percent changes of −33% and − 18%, respectively [15]. Therefore, the minimum required sample size was 85 patients in each group.

### Study population

Following approval by our institutional ethics committee and registration of the study (NCT 06818383), we retrospectively checked our database for patients who underwent Rezūm therapy at our center from January 2021 to January 2025. One hundred seventy patients were included and divided into two equal groups: small (< 80 g) and large (80–120 g) prostate groups. We recorded the relevant information including age, prostatic size, median lobe enlargement, preoperative catheter dependence, prostate-specific antigen (PSA), post-void residual (PVR), maximum urinary flow rate (Qmax), International Prostate Symptom Score (IPSS), quality of life (QoL), International Index of Erectile Function (IIEF), number of vapor injections, operative time, catheter duration and hospital stay. Primary outcome was to assess efficacy and safety of Rezūm therapy in the 2 groups through comparison of the pre- and 3, 12-, 24-, 36- & 48-months post-procedural Qmax, IPSS, PVR, QoL, PSA, IIEF, prostate size and complications.

### Technique

The procedure was performed as a day-case under general anesthesia or a combination of sedation and local anesthesia by the same expert team of urologists. A single-use needle was inserted into the prostatic tissue for 9 s. The total number of injections was determined based on the size and shape of the prostate, including both the lateral and median lobes. Postoperative urethral catheterization protocols varied according to the total number of injections and the duration of preoperative urinary retention.

### Statistical methods

The Student’s t-test was used to assess the statistical significance of differences between two means. The Mann-Whitney U test was employed to compare non-parametric variables between two study groups. Post hoc tests were used for multiple comparisons of group means. Chi-square or Fisher’s exact test were applied to evaluate relationships between qualitative variables. All data were reviewed and analyzed using the Statistical Package for the Social Sciences (SPSS) version 27. A p-value of < 0.05 was considered statistically significant.

## Results

A total of 170 patients who underwent Rezūm were included. Eighty-five patients had a small prostate (< 80 g), and 85 had a large prostate (80–120 g). The demographic data were comparable between groups, except for prostate size and PSA, both of which were significantly higher in the large prostate group (*p* < 0.001) (Table 1).

**Table 1:** Preoperative characteristics of patients

	Group		*p*-value
	Small prostate	Large prostate	
	Mean ± SD	Mean ± SD	
Age (Year)	65.74 ± 7.4	64.56 ± 8.61	0.341
Prostatic size (gm)	64.98 ± 8.51	99.59 ± 12.05	< 0.001
Median lobe presence	24 (28.24%)	30 (35.29%)	0.323
Preoperative urinary retention	20 (23.53%)	28 (32.94%)	0.173
PSA (ng/mL)	4.03 ± 0.96	5.16 ± 0.83	< 0.001
PVR (mL)	98.78 ± 24.94	102.67 ± 23.1	0.292
Qmax (mL/s)	9.26 ± 1.1	9.09 ± 1.64	0.443
IPSS	19.33 ± 2.41	19.68 ± 2.44	0.344
QoL	4.56 ± 0.79	4.94 ± 0.75	0.002
IIEF	14.72 ± 6.08	13.14 ± 4.97	0.066

The mean number of vapor injections was significantly higher in the large prostate group (7.7 ± 1.0) compared to the small prostate group (4.09 ± 1.2) (*p* < 0.001). Operative time and catheter duration were significantly longer in the large prostate group (*p* < 0.001), while hospital stay was similar between groups (*p* = 0.407) (Table 2).

**Table 2:** Perioperative data

	Group		*p*-value
	Small prostate	Large prostate	
	Median (IQR)	Median (IQR)	
Operative time (min)	7 (6–8)	12 (10–14)	< 0.001
Hospital stay (days)	1 (1–1)	1 (1–1)	0.407
Catheter duration (days)	6 (5–7)	9 (9–11)	< 0.001

At 48 months post-Rezūm, both groups demonstrated statistically significant improvement in all measured outcomes (Qmax, IPSS, PVR, QoL, PSA, IIEF, and prostate size) (*p* < 0.05) (Table 3).

**Table 3:** Clinical outcomes over time

	Group				*p*-value
	Small prostate		Large prostate		
	Mean ± SD	mean % of change	Mean ± SD	mean % of change	
**Qmax (mL/s)**					
Baseline	9.26 ± 1.1		9.09 ± 1.64		0.443
3 months	13.53 ± 1.41	46.86%	12.31 ± 1.92	36.19%	< 0.001
12 months	14.21 ± 1.42	54.44%	12.84 ± 1.56	43.21%	< 0.001
24 months	13.93 ± 1.56	51.44%	12.75 ± 1.98	41.12%	< 0.001
36 months	13.59 ± 1.61	47.89%	12.72 ± 1.92	40.97%	0.002
48 months	13.95 ± 1.43	51.66%	12.47 ± 1.74	38.49%	< 0.001
**IPSS**					
Baseline	19.33 ± 2.41		19.68 ± 2.44		0.344
3 months	9.95 ± 1.49	−48.43%	12.14 ± 1.94	−38.27%	< 0.001
12 months	9.69 ± 1.33	−49.42%	11.46 ± 1.63	−41.39%	< 0.001
24 months	9.74 ± 1.41	−49.44%	11.98 ± 1.75	−38.89%	< 0.001
36 months	9.93 ± 1.45	−48.24%	11.87 ± 1.7	−39.52%	< 0.001
48 months	9.89 ± 1.42	−48.44%	11.69 ± 1.62	−40.23%	< 0.001
**PVR (mL)**					
Baseline	98.78 ± 24.94		102.67 ± 23.1		0.292
3 months	53.35 ± 13.37	−45.40%	71.98 ± 17.37	−29.92%	< 0.001
12 months	46.59 ± 10.36	−51.47%	64.18 ± 13.86	−37.00%	< 0.001
24 months	50.67 ± 14.68	−47.84%	69.68 ± 16.98	−32.10%	< 0.001
36 months	53.18 ± 11.36	−44.94%	69.72 ± 17.57	−32.17%	< 0.001
48 months	50.18 ± 11.61	−48.62%	66.47 ± 14.86	−35.09%	< 0.001
**QoL**					
Baseline	4.56 ± 0.79		4.94 ± 0.75		0.002
3 months	2.98 ± 0.44	−33.67%	3.28 ± 0.61	−33.29%	< 0.001
12 months	2.13 ± 0.48	−53.08%	3.01 ± 0.59	−38.71%	< 0.001
24 months	2.16 ± 0.55	−52.67%	3.27 ± 0.62	−33.59%	< 0.001
36 months	2.21 ± 0.6	−51.75%	3.04 ± 0.57	−38.24%	< 0.001
48 months	2.2 ± 0.51	−51.76%	3.06 ± 0.56	−37.75%	< 0.001
**PSA (ng/mL)**					
Baseline	4.03 ± 0.96		5.16 ± 0.83		< 0.001
3 months	2.76 ± 0.67	−30.80%	3.56 ± 0.62	−29.99%	< 0.001
12 months	2.8 ± 0.7	−29.50%	3.18 ± 0.81	−37.29%	0.001
24 months	2.88 ± 0.73	−27.83%	3.47 ± 0.57	−31.62%	< 0.001
36 months	2.76 ± 0.67	−30.92%	3.48 ± 0.5	−31.59%	< 0.001
48 months	2.75 ± 0.66	−30.99%	3.59 ± 0.5	−29.70%	< 0.001
**IIEF**					
Baseline	14.72 ± 6.08		13.14 ± 4.97		0.066
3 months	12.31 ± 2.76	−2.09%	12.81 ± 4.94	−1.59%	0.411
12 months	15.82 ± 5.55	13.08%	13.85 ± 4.82	6.77%	0.014
24 months	15.6 ± 5.56	10.63%	13.28 ± 4.79	3.07%	0.004
36 months	15.55 ± 5.83	9.30%	13.25 ± 5.02	1.83%	0.006
48 months	15.55 ± 5.83	9.30%	13.6 ± 4.92	4.17%	0.019
**Prostate size (g)**					
Baseline	64.98 ± 8.51		99.59 ± 12.05		< 0.001
12 months	45.33 ± 6.21	−29.99%	70.67 ± 8.3	−28.78%	< 0.001
24 months	46.92 ± 7.07	−27.77%	72.99 ± 9.56	−26.75%	< 0.001
36 months	46.92 ± 7.07	−27.77%	73.99 ± 8.26	−25.56%	< 0.001
48 months	45.92 ± 6.25	−29.15%	72.32 ± 8.07	−27.22%	< 0.001

Regarding postoperative complications, there were no significant differences between the groups except for the need for retreatment. Retreatment was required in 2 patients (2.35%) with small prostates and 13 patients (15.29%) with large prostates, which was statistically significant (*p* = 0.003) (Table 4).

**Table 4:** Postoperative complications

		Group		p-value
		Small prostate	Large prostate	
		N (%)	N (%)	
Hematuria	No	83 (97.65%)	81 (95.29%)	0.682
	Yes	2 (2.35%)	4 (4.71%)	
Dysuria	No	65 (76.47%)	57 (67.06%)	0.173
	Yes	20 (23.53%)	28 (32.94%)	
UTI	No	77 (90.59%)	77 (90.59%)	1.00
	Yes	8 (9.41%)	8 (9.41%)	
Urine retention	No	81 (95.29%)	77 (90.59%)	0.231
	Yes	4 (4.71%)	8 (9.41%)	
Retrograde ejaculation	No	83 (97.65%)	85 (100%)	0.497
	Yes	2 (2.35%)	0 (0%)	
Urgency incontinence	No	81 (95.29%)	79 (92.94%)	0.514
	Yes	4 (4.71%)	6 (7.06%)	
Retreatment need	No	83 (97.64%)	72 (84.7%)	0.003
	Yes	2 (2.35%)	13 (15.29%)	
Hematospermia	No	81 (95.29%)	84 (98.82%)	0.368
	Yes	4 (4.71%)	1 (1.18%)	

In patients with preoperative urinary retention, our protocol was an initial trial of voiding at 1 week then every week for a maximum period of 4 weeks. Also, we considered keeping urethral catheter 1 day for every 10gm prostate size. In the large prostate group, 6 patients failed to void after catheter removal. Most of retreatment need was in the first 2 years in both groups (Table 5). Our retreatment strategy was:

- Mild symptoms: medical treatment with alpha-blockers was the initial strategy.
- Moderate symptoms or unfit for general anesthesia: repeat Rezūm therapy was chosen.
- Severe symptoms or failed TWOC: B-TURP was the preferred retreatment option.

**Table 5:** Retreatment rates

	1 st year	2nd year	3rd year	4th year
Small prostate No. (%)	2 (2.3%)	0	0	0
Large prostate No. (%)	8 (9.4%)	4 (4.7%)	1 (1.1%)	0

## Discussion

Benign prostatic hyperplasia is a pathological condition characterized by the proliferation of epithelial and smooth muscle cells, leading to an increase in prostate size. BPH is a common health condition that causes bothersome storage and voiding lower urinary tract symptoms (LUTS). Furthermore, the severity of BPH increases proportionally with age [16, 17]. The healthcare system experiences a significant burden from LUTS/BPH, which is among the top 10 most costly male diseases [18, 19].

Treatment alternatives for patients with BPH of large gland size are B-TURP, HOLEP, and laparoscopic or open prostatectomy [20–22]. Meanwhile, most treatment guidelines recommend Rezūm therapy for prostates sized 30–80 g, as only a limited number of studies have investigated its role in managing larger prostates [13].

Regarding perioperative outcomes, the large prostate group in our study had a median operative time of 12 (10–14) minutes and a median post-procedure catheter duration of 9 (9–11) days. In contrast, Elterman et al. reported a median operative time of 5.5 (4.9–6.6) minutes and a median catheterization duration of 9 (7–14) days [12].

In terms of treatment efficacy, our study demonstrated that both the small and large prostate groups showed statistically significant improvements in Qmax, IPSS, PVR, QoL, PSA, IIEF, and prostate size at 48 months post-Rezūm. Similarly, Bole et al. found comparable improvements in clinical outcomes at 3 months post-Rezūm between the two groups in terms of Qmax, AUA symptom score, and PVR [14]. Additionally, Elterman et al., in their multicenter study, reported that patients with large prostates experienced early and sustained improvements after one year [12]. Conversely, Garden et al. found that patients with large prostates had significant improvements in PVR and Qmax, but not in the Sexual Health Inventory for Men (SHIM) or AUA symptom score. In contrast, patients with small prostates showed improvements in PVR and AUA symptom score, but not in Qmax [15].

In the present study, postoperative complications were comparable between the two groups, except for the need for retreatment, which was significantly higher in the large prostate group. Among patients in the small prostate group, six continued alpha-blocker therapy, one underwent repeat Rezūm, and one underwent B-TURP. In the large prostate group, five continued alpha-blockers, two underwent repeat Rezūm, and eleven underwent B-TURP. Similarly, Bole et al. reported no significant difference in complication rates between small and large prostate groups [14]. In contrast, Garden et al. found that while complications were generally mild, post-procedure UTI was significantly higher in the large prostate group (*p* = 0.002)^15^.

Study limitations include its retrospective nature and lack of randomization, as well as the absence of median lobe size estimation, which may influence Rezūm outcomes. Additionally, the study compared outcomes with a control group that underwent Rezūm for small prostates rather than an alternative treatment modality such as HoLEP or B-TURP. This limitation can be attributed to the fact that Rezūm is more preferred for patients with significant medical comorbidities who are risky for more invasive options making proper randomization difficult. Nevertheless, we believe this is the first study to evaluate the long-term durability of Rezūm therapy in patients with large prostates.

## Conclusion

Rezūm therapy is a safe and effective treatment option for BPH across a wide range of prostate sizes. However, patients with larger prostates may experience a higher rate of retreatment during long-term follow-up. Further prospective and comparative studies are warranted to validate our findings, particularly in relation to alternative surgical options for managing large prostates.

## Data Availability

No datasets were generated or analysed during the current study.
